# The Reaction between K_2_CO_3_ and Ethylene Glycol in Deep Eutectic Solvents

**DOI:** 10.3390/molecules29174113

**Published:** 2024-08-30

**Authors:** Yi Zhou, Mingzhe Chen, Xueling Dong, Dezhong Yang

**Affiliations:** School of Science, China University of Geosciences, Beijing 100083, China; 2019230040@email.cugb.edu.cn (Y.Z.); 2019220039@email.cugb.edu.cn (M.C.)

**Keywords:** alcohols, carbon dioxide, eutectic solvents, hydrogen bonds, potassium carbonate

## Abstract

Understanding intermolecular interactions is important for the design of deep eutectic solvents. Herein, potassium carbonate (K_2_CO_3_) and ethylene glycol (EG) are used to form deep eutectic solvents. The interactions between K_2_CO_3_ and EG are studied using nuclear magnetic resonance (NMR) and Fourier transform infrared (FTIR) spectra. Interestingly, the interaction results indicate that the carbonate anion ${\mathrm{CO}}_{3}^{2-}$ can react with EG to form EG-based organic carbonate, which can occur even at room temperature. The possible reaction steps between K_2_CO_3_ and EG are presented. As K_2_CO_3_ can be prepared from CO_2_ and KOH, the findings of this work may provide a promising strategy for CO_2_ capture and conversion.

## 1. Introduction

Over the last few decades, deep eutectic solvents (DESs) have been developed and received a lot of attention mainly because of their advantageous characteristics, such as their low volatility, low flammability, simple preparation procedures, and structure tunability [1,2]. Generally, DESs are described as a kind of fluid consisting of at least two or more components, while they present lower melting points than each parent component [3,4]. To date, DESs can be classified into several types according to their components, and many DESs are prepared by mixing hydrogen bond donors (HBDs) and hydrogen bond acceptors (HBAs) [5,6,7,8]. Due to their attractive properties, the applications of DESs have been explored in many fields, including organic reactions, biomass treatment, separations and extractions, electrochemistry and batteries, and gas capture [9,10,11,12].

DESs based on potassium carbonate (K_2_CO_3_) are also explored mainly because K_2_CO_3_ is a common and cheap inorganic base with environmentally benign characteristics [13,14]. K_2_CO_3_-based DESs are already used in lignocellulose pretreatment, electrode material separation, supercapacitors, and gas separations [15,16,17,18,19,20,21,22]. Among these K_2_CO_3_-based DESs, K_2_CO_3_-EG DESs (EG: ethylene glycol) have received significant attention, as EG is also a cheap and renewable compound. The physiochemical properties of K_2_CO_3_-EG have been thoroughly investigated [14,22], and the interactions between K_2_CO_3_ and EG have been studied using theoretical calculations [16,19]. The theoretical results suggest that intermolecular hydrogen bonds formed between the -OH hydrogen of EG and the carbonate anion of K_2_CO_3_.

In this work, the interactions between K_2_CO_3_ and EG in DESs were further studied using NMR and FTIR spectra. Surprisingly, the NMR results reveal that the ${\mathrm{CO}}_{3}^{2-}$ anion can react with EG to form EG-based carbonate species, which was not disclosed by the previous studies of K_2_CO_3_-EG DESs reported in the literature. The results can be found in the following sections. 

## 2. Results and Discussion

**Figure 1:**
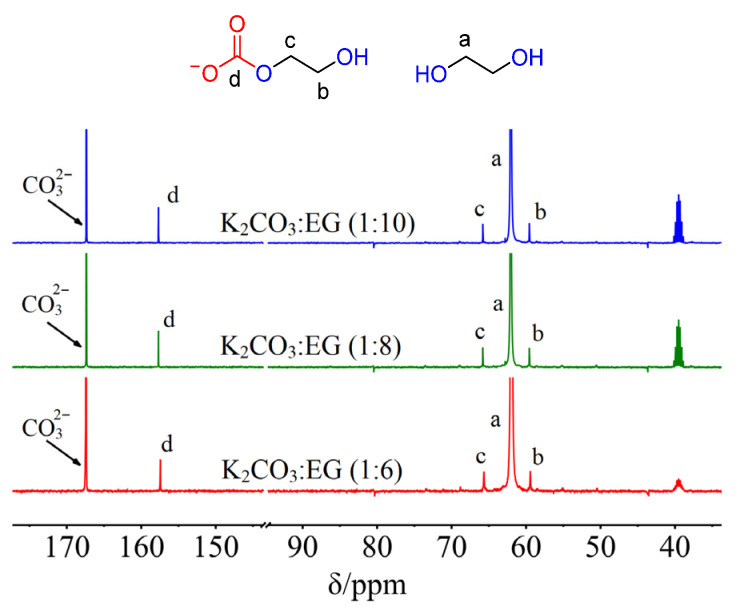
The ^13^C NMR spectra of K_2_CO_3_-EG DESs. Letters a–d are labels of carbons of EG and EG-based carbonate.

**Figure 2:**
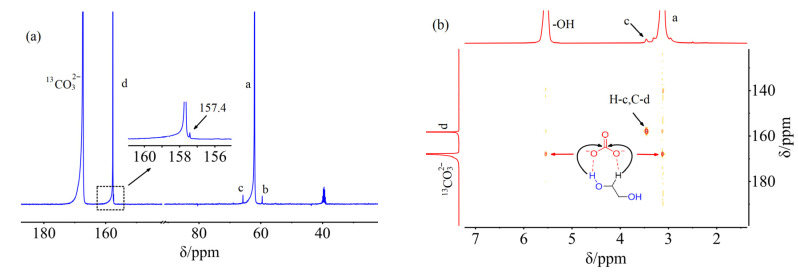
The ^13^C NMR (**a**) and ^1^H-^13^C HMBC (**b**) spectra of K_2_^13^CO_3_:EG (1:8). Letters a–d are labels of carbons or hydrogens of EG and EG-based carbonate.

At first, three DESs K_2_CO_3_:EG (1:6), (1:8), and (1:10) were prepared. The ^13^C NMR spectra of these K_2_CO_3_-EG DESs were recorded using DMSO-*d*_6_ as an external solvent. Namely, the interactions between K_2_CO_3_ and EG were not disturbed by DMSO-*d*_6_. As shown in Figure 1, there are three new carbon peaks C-b, C-c, and C-d besides the ${\mathrm{CO}}_{3}^{2-}$ carbon and -CH_2_- carbon (C-a) of EG for each K_2_CO_3_-EG used. The three new peaks can be found at 59.6 (C-b), 65.8 (C-c), and 157.7 (C-d) ppm for K_2_CO_3_:EG (1:8). The new peaks C-b and C-c are attributed to the -CH_2_- carbons of HO-CH_2_-CH_2_-O-COO^−^ carbonate, and the peak C-d is attributed to the carbonyl carbon of HO-CH_2_-CH_2_-O-COO^−^ [23,24,25]. The ^13^C NMR spectra of K_2_^13^CO_3_:EG (1:8) were also investigated. As shown in Figure 2a, the carbon peaks C-b, C-c, and C-d can be observed. The C-d peak in K_2_^13^CO_3_:EG (1:8) is significantly enhanced relative to that in K_2_CO_3_:EG (1:8), suggesting that C-d carbon comes from K_2_^13^CO_3_. In other words, C-d carbon in K_2_CO_3_-EG DESs is from the ${\mathrm{CO}}_{3}^{2-}$ carbon of K_2_CO_3_. Interestingly, there is another weak peak at 157.4 ppm (Figure 2a), which can be ascribed to the carbonyl carbon of the dianion ^−^OOC-O-CH_2_-CH_2_-O-COO^−^ [26]. This peak is not detected in K_2_CO_3_-EG systems, which may be due to its low concentration in K_2_CO_3_-EG DESs.

Furthermore, the ^1^H-^13^C HMBC spectra of K_2_^13^CO_3_:EG (1:8) were recorded to demonstrate the interactions between K_2_^13^CO_3_ and EG. As can be seen in Figure 2b, there is a cross-signal between H-c and C-d, confirming the formation of HO-CH_2_-CH_2_-O-^13^COO^−^ carbonate. Moreover, the correlations between ^13^${\mathrm{CO}}_{3}^{2-}$ carbon and the -OH proton or -CH_2_- proton can be found, suggesting that both the -OH proton and -CH_2_- proton in EG can form hydrogen bonds with the O atom of ${\mathrm{CO}}_{3}^{2-}$ in K_2_CO_3_-EG DESs. However, the hydrogen bonds between the -CH_2_- proton and ${\mathrm{CO}}_{3}^{2-}$ in K_2_CO_3_-EG DESs were not revealed in previous reports [16,19].

**Figure 3:**
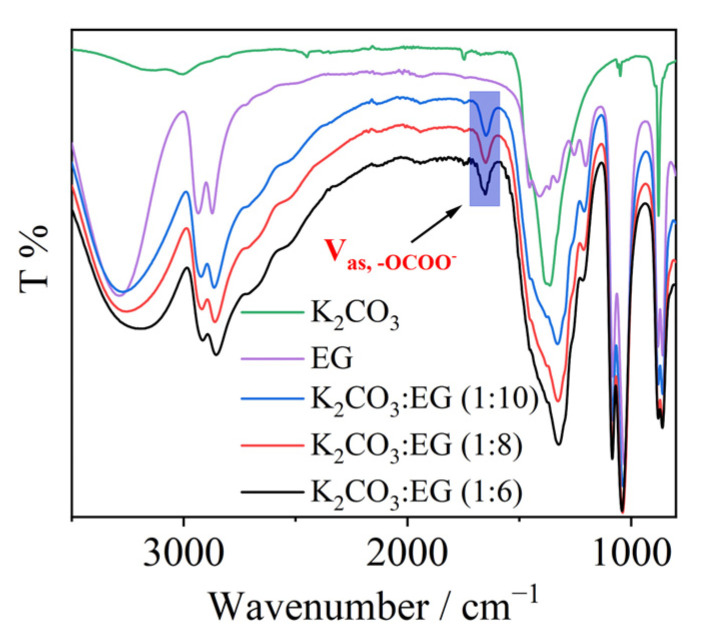
The FTIR spectra of K_2_CO_3_, EG, and K_2_CO_3_-EG DESs.

Figure 3 presents the FTIR spectra of K_2_CO_3_, EG, and K_2_CO_3_-EG used in this work. In comparison with the FTIR spectra of K_2_CO_3_ and EG, new bands can be observed at ~1650 cm^−1^ in the spectra of K_2_CO_3_-EG. The band at ~1650 cm^−1^ is ascribed to the C=O asymmetrical stretching mode of R-OCOO^−^ [27,28]. The FTIR results again suggest the formation of EG-based carbonate. The new bands for K_2_CO_3_:EG (1:6), (1:8), and (1:10) are at 1653, 1651, and 1650 cm^−1^, respectively.

Based on the above spectral results, the reaction between K_2_CO_3_ and EG can be elucidated, which may proceed in the following steps.


(1)

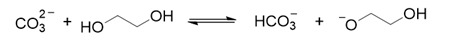




(2)

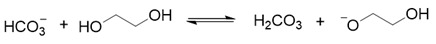




(3)

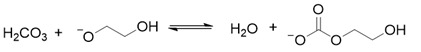




(4)

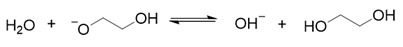



The overall reaction is shown in Equation (5).


(5)

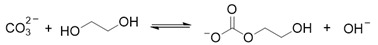



As seen in Figure 2a, the formation of the dianion ^−^OOC-O-CH_2_-CH_2_-O-COO^−^ can be detected, so the reaction shown in Equation (6) can occur in K_2_CO_3_-EG DESs.


(6)

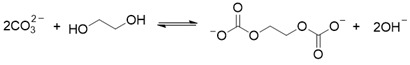



It is worth noting that the signal of the dianion ^−^OOC-O-CH_2_-CH_2_-O-COO^−^ is much weaker compared to that of HO-CH_2_-CH_2_-O-COO^−^ in Figure 2a, i.e., the main product of the reaction between K_2_CO_3_ and EG is HO-CH_2_-CH_2_-O-COO^−^. Therefore, the main reaction between K_2_CO_3_ and EG can be represented by Equation (5). Moreover, the pH values of K_2_CO_3_:EG (1:6) and (1:8) were 12.73 and 13.2 at 30 °C [14], respectively, which implied the formation of the OH^−^ anion in K_2_CO_3_-EG solvents and supported the reaction shown by Equations (5) and (6). The aforementioned results reveal that the reaction occurs between K_2_CO_3_ and EG, forming alcohol-based carbonates, suggesting that the reported theoretical calculations for the interactions between K_2_CO_3_ and EG are not accurate [16,19].

All the three DESs K_2_CO_3_:EG (1:6), (1:8), and (1:10) can be formed by heating K_2_CO_3_-EG mixtures at 80 °C and 1.0 atmosphere. It should be noted that K_2_CO_3_:EG (1:10) can also be easily prepared by mixing K_2_CO_3_ and EG at room temperature, and the peaks of EG-based carbonate can still be found in the NMR and FTIR spectra of K_2_CO_3_:EG (1:10) prepared at room temperature. In other words, the reaction between K_2_CO_3_ and EG could proceed at room temperature. Moreover, as we all know, K_2_CO_3_ is a common and cheap inorganic base, which can be produced through the reaction between KOH and CO_2_. Therefore, the findings of our work might be beneficial to developing new pathways for CO_2_ capture and conversion.

## 3. Materials and Methods

### 3.1. Materials and Characterizations

EG (99.5%) was purchased from J&K Scientific Ltd. (Beijing, China). K_2_CO_3_ and K_2_^13^CO_3_ were obtained from Innochem (Beijing, China). EG was dried by a 4 Å molecular sieve prior to use, and K_2_CO_3_ and K_2_^13^CO_3_ were dried by a vacuum pump. N_2_ (99.999%) was obtained from Beijing ZG Special Gases Sci. and Tech. Co., Ltd. (Beijing, China).

FTIR spectra were recorded on a Nicolet 6700 spectrometer (Waltham, MA, USA) with an attenuated total reflection (ATR) accessory. ^1^H NMR (400 MHz) and ^13^C NMR (100.6 MHz) spectra were obtained on a Bruker spectrometer (Bruker Biospin, Karlsruhe, Germany) and DMSO-*d*_6_ was used as the reference. 

### 3.2. Synthesis of DESs

Each K_2_CO_3_-EG DES was prepared by mixing K_2_CO_3_ and EG at the desired molar ratio in a round flask (10 mL) under N_2_ atmosphere, and the mixture was stirred at 80 °C until a homogenous solution was formed. 

For K_2_CO_3_:EG (1:10) DESs, they can also be obtained by stirring K_2_CO_3_ and EG at room temperature. 

### 3.3. NMR and FTIR Data

NMR data:

K_2_CO_3_:EG (1:6): ^13^C NMR (100.6 MHz, DMSO-*d*_6_, ppm) δ = 59.4, 61.9, 65.7, 157.4, 167.4.

K_2_CO_3_:EG (1:8): ^13^C NMR (100.6 MHz, DMSO-*d*_6_, ppm) δ = 59.6, 62.1, 65.8, 157.7, 167.4.

K_2_CO_3_:EG (1:10): ^13^C NMR (100.6 MHz, DMSO-*d*_6_, ppm) δ = 59.6, 62.8, 65.8, 157.7, 167.4.

FTIR data:

K_2_CO_3_:EG (1:6): 3188, 2915, 2855, 1653, 1324, 1085, 1041, 880, 861, 516 cm^−1^.

K_2_CO_3_:EG (1:8): 3256, 2918, 2861, 1651, 1328, 1084, 1039, 880, 860, 515 cm^−1^.

K_2_CO_3_:EG (1:10): 3273, 2923, 2864, 1650, 1329, 1083, 1037, 880, 860, 514 cm^−1^.

## 4. Conclusions

In summary, K_2_CO_3_ can react with EG in K_2_CO_3_-EG DESs, resulting in the formation of EG-based organic carbonate HO-CH_2_-CH_2_-O-COO^−^ as the main product, and the reaction can occur at room temperature. The HMBC NMR results disclose that both the -OH and -CH_2_- hydrogens form hydrogen bonds with the O atom of K_2_CO_3_. The transformation of carbon from K_2_CO_3_ to EG might bring valuable information for the development of CO_2_ capture and conversion technologies.

## Data Availability

The data used in this work are available on request from the corresponding authors.
